# Brigadier General Charles Ravenscroft Greenleaf

**Published:** 1911-11

**Authors:** 


Brigadier General Charles Ravenscroft Greenleaf, Medical College of Ohio, 1860, a military surgeon from the outbreak of the Civil War, through the Spanish American War, and till his retirement in 1902, died in San Jose, Cal., September 3, 1911, aged 73. He was a surgeon and executive of ability, a writer of note, especially on military topics.

